# Prevalence of Depression and Sleep Disorders in Patients on Dialysis: A Cross-Sectional Study in Qatar

**DOI:** 10.1155/2021/5533416

**Published:** 2021-05-26

**Authors:** Fadwa Al-Ali, Mostafa Elshirbeny, Abdullah Hamad, Ahmad Kaddourah, Tarek Ghonimi, Rania Ibrahim, Tarek Fouda

**Affiliations:** Department of Nephrology, Hamad Medical Corporation, Doha, ZIP/Post Code 3050, Qatar

## Abstract

Patients with end-stage renal disease treated with dialysis have poor quality of life (QOL). Improving QOL in these patients with multiple comorbidities is a large challenge. We performed a cross-sectional study to evaluate the prevalence and associated factors of depression and sleep disorders in this population. Our primary aim was to evaluate QOL measures in dialysis patients in Qatar through a series of validated questionnaires mainly concerning depression and sleep disorders. Our secondary aim was to study the associations of age, sex, and comorbid conditions with the QOL measures. We hypothesized that end-stage renal disease (ESRD) patients on dialysis would have disturbed QOL due to both ESRD and dialysis and comorbidities. This prospective cross-sectional study included adult ESRD patients receiving either hemodialysis (HD) or peritoneal dialysis (PD) in the main tertiary dialysis unit in Qatar. We administered two surveys to evaluate depression (the Center for Epidemiologic Studies Depression Scale, http://www.bmedreport.com/archives/7139) and sleep disorders (the Pittsburgh Sleep Quality Index, https://www.sleep.pitt.edu/instruments/). We also reviewed patient demographics, comorbidities, and laboratory test results to evaluate any associated factors. We randomly studied 253 patients (62% on HD and 38% on PD). Overall, 48% of patients had depression, while 83.8% had sleep disorders. The PD had more poor sleepers than the HD group (89.1% versus (vs.) 75%, *p*=0.003). Most of our dialysis patients had poor sleep, but it was more significant in the elderly group 109 (90%) than in the young group 103 (78%) (*p*=0.009). Patients with diabetes mellitus (DM) had significantly more prevalence of poor sleep (131 (88.5%)) than those without DM (81 (77.1%), *p*=0.01). More female patients had depression than male patients (52% vs. 25%, *p* < 0.0001; odds ratio: 3.27 (95% confidence interval: 1.9–5.6), *p* < 0.0001). This is the first study in Qatar to evaluate depression and sleep disorders in patients on dialysis therapy.

## 1. Introduction

Patients with end-stage renal disease (ESRD) who are treated with dialysis experience many risks to their health-related quality of life (HRQOL), both from the myriad symptoms of ESRD itself and from the physical and mental burden of dialysis treatment. For these patients, the careful assessment of HRQOL can help guide the provision of medical management to optimize their health experience [1].

In ESRD, low quality of life (QOL) is associated with increased morbidity and mortality. The modality of renal replacement therapy may affect QOL in ESRD, although previous studies comparing QOL in patients on continuous ambulatory peritoneal dialysis (CAPD) and hemodialysis (HD) showed conflicting results [2].

It is widely accepted that ESRD patients experience various problems due to medical illness as well as psychological problems that exert a negative influence on the outcome of the disease and QOL. Anxiety and depression are the most common psychiatric disorders that coincide with renal failure [3]. Depression contributes significantly to a diminished QOL in this population and can have a negative impact on patients' nutritional status and their compliance with dialysis treatment [4].

In patients with ESRD, sleep disorders usually present as insomnia, restless leg syndrome, obstructive sleep apnea syndrome, or sleep apnea-hypopnea syndrome, excessive daytime sleepiness, narcolepsy, sleepwalking, nighttime waking, nightmares, rapid eye movement behavioral disorder, periodic limb movements in sleep, and poor concentration, which are sometimes ascribed to the uremic state itself [5]. However, improvements in the uremic state by either dialysis or renal transplant may not necessarily ameliorate the sleep disorders. This indicates that more complicated processes that cause sleep disorders in patients with ESRD are not fully understood yet [6].

QOL is becoming an important outcome measure after the initiation of renal replacement therapy. The major therapeutic goal is to improve the functioning ability of these patients so that they can enjoy life to their fullest. Herein, we aimed (1) to assess the prevalence of depression and sleep disorders in dialysis patients in Qatar for the first time and (2) to assess the risk factors (i.e., age, sex, and comorbid conditions) correlated with both disorders and determine which group of dialysis patients requires more screening and therapeutic interventions to solve these two major health issues. We hypothesized that ESRD patients on dialysis would have disturbed QOL due to both ESRD and dialysis and comorbidities.

## 2. Materials and Methods

### 2.1. Ethics Statements

The study was approved by our institutional review board committee of Hamad General Hospital. All patients provided informed consent before study participation.

#### 2.1.1. Study Design, Setting, and Population

We conducted a cross-sectional study in patients with ESRD who received either HD or peritoneal dialysis (PD) at the main tertiary dialysis unit in Doha, Qatar, which serves about 900 dialysis patients. The inclusion criteria were patients aged 18 years or older, those treated at an in-center dialysis clinic, and those who received chronic maintenance HD or PD for at least 1 month. The exclusion criteria were patients younger than 18 years of age and those treated with a home-based dialysis modality or receiving HD for acute renal failure.

#### 2.1.2. Study Procedures and Data Collection

**Figure 1:**
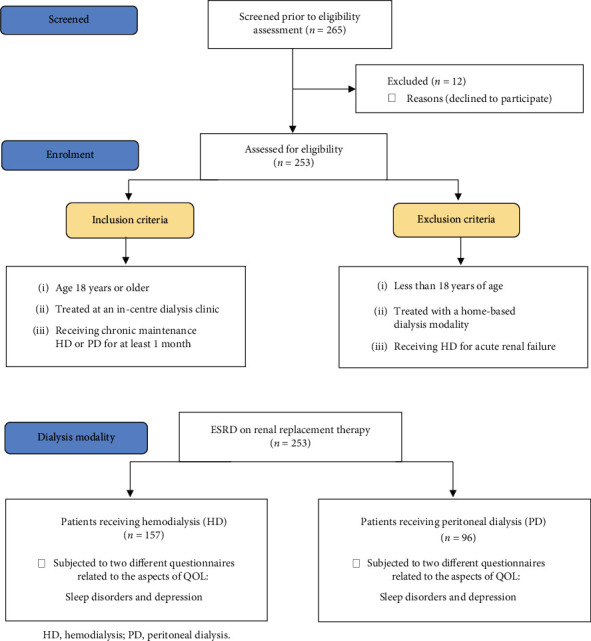
Study flow diagram.

To address our primary objective, we asked all participants to complete two different validated questionnaires related to the aspects of QOL (sleep disorders and depression) (Figure 1). As we explained to the patients, the questionnaires are mainly used for assessing sleep disorders. Because of the participants' health condition and continuous visits for dialysis treatment, we thought the questionnaire was suitable as a data collection instrument since ESRD patients with multiple comorbidities commonly complain of sleep disorders, mood swings, and decreased daily life activity.

The following questionnaires were used to evaluate our study objectives:

1. The Center for Epidemiologic Studies Depression Scale (CES-D) is a screening test for depression and depressive disorder that consists of 20 items. A score of 16 or more is enough for the diagnosis of depression.
2. The Pittsburgh Sleep Quality Index (PSQI) is an effective instrument used to measure the quality and patterns of sleep in adults and to differentiate the quality of sleep (poor versus good) and its pattern in adults. The PSQI has a sensitivity of 89.6% and a specificity of 86.5% for identifying cases with sleep disorders, using a cutoff score of 5 [7].

We also recorded the patients' demographics, diagnosis, and laboratory data including glucose, hemoglobin, albumin, calcium, phosphate, parathyroid hormone (PTH), glycated hemoglobin (HbA1c) levels, and adequacy of dialysis.

To address our secondary objective, we reviewed the patient's characteristics and laboratory and diagnostic investigations for each patient through our data system and our quality unit. Patients' characteristics including demographic and nondemographic data were collected and analyzed as follows:

1. Demographic and clinical characteristics included age, sex, ethnic group, nationality, smoking status, nutritional status, dialysis adequacy (single-pool Kt/V), type of vascular access, vintage (time on dialysis), type of membrane in patients with PD, and comorbidities such as the presence of diabetes, hypertension, infections, hepatitis, and cardiovascular diseases.
2. Nondemographic characteristics included both laboratory and diagnostic investigations. Laboratory investigations included levels of the complete blood count, PHT, calcium, phosphorus, HbA1c, iron, vitamin D level, serum albumin, electrolytes, and lipid profiles. Diagnostic investigations included electrocardiography, echocardiography, and chest radiography.

#### 2.1.3. Sample Size and Sampling Technique

Patients were enrolled in the study using a nonprobability convenience sampling technique. The sample size was calculated using a cross-sectional study sample size calculation technique with a 95% confidence level and 5% margin of error [8]. The minimum effective sample size required was 248 patients with ESRD. The sample of the whole population of patients with ESRD was based on the total number and distribution of patients at the Fahad Bin Jassim Kidney Center (HD = 533 and PD = 170; total = 703). The proportions of patients with HD and those with PD were 75.8% and 24.18%, respectively. Thus, the numbers needed in each category based on the calculated proportions were 223 patients with HD and 117 patients with PD.

### 2.2. Statistical Analysis

Continuous variables are presented as mean ± standard deviation or median and range, and categorical variables are presented as absolute and relevant frequencies. To compare parametric variables between the groups, the paired *T*-test was used, and to compare nonparametric variables between the groups, the chi-square test was used. Multiple logistic regression analysis was performed to assess the association of several demographic and clinical characteristics with mortality in our patients. We report the adjusted odds ratios with 95% confidence intervals (CIs) herein. Analyses of the results were performed using Statistical Package for Social Sciences version 17.0 for Windows (IBM Corp., Armonk, NY). Probability values of *p* < 0.05 (two-tailed) were considered statistically significant. No missing data were reported as we used a nonprobability convenience sampling technique.

## 3. Results

### 3.1. Demographic Data and Comorbidities

In this study, 253 ESRD patients on renal replacement therapy were enrolled; 157 (62%) patients were on HD, and 96 patients were on PD (38%). Patients' mean age was 56 ± 14 years. There were 137 female patients (54%) and 116 male patients (46%). Their mean weight was 76 ± 26 kg, and mean body mass index (BMI) was 29 ± 7 kg/m^2^.

Overall, 148 (58.5%) patients had DM. The most common causes of ESRD were DM (146 patients (57%)) and hypertension (81 patients (32%)).  Table 1 shows the demographic data and laboratory test results of the study population.

### 3.2. General Quality of Life Outcomes

One hundred twenty-two (48%) patients had depression (62 with mild depression (CES-D score ≤21) and 60 with moderate-to-severe depression (CES-D score >21)). Univariate regression analysis revealed no significant correlation between CES-D and the BMI, serum albumin level, vitamin D level, PTH level, serum calcium level, phosphate level, or Kt/V level. Two hundred twelve patients had poor sleep (PSQI score ≥5). The median PSQI score was 10 (interquartile range (IQR): 6–18).  Table 1 shows the general QOL outcomes regarding depression and sleep disorders.

### 3.3. Quality of Life Outcomes: Diabetic versus Nondiabetic Patients

Of 253 patients, 148 (58.5%) patients had DM, and 105 (41.5%) patients did not. Seventy-eight patients had depression (52.7%) (DM group), whereas 44 patients (41.9%) did not (non-DM group, *p*=0.09).

Diabetic patients had a significantly higher median PSQI score of 10 (IQR: 7–18.75) than nondiabetics (median: 9, IQR: 5–16.5) (*p*=0.02). The DM group had significantly more patients with poor sleep (131 (88.5%)) than the non-DM group (81 (77.1%), *p*=0.01). Univariate analysis of other clinical markers including the hemoglobin level, anemia, PTH level, Kt/V level, presence of hypertension, and phosphorus level revealed no statistical correlation with the PSQI scores (Table 2).

### 3.4. Quality of Life Outcomes: Peritoneal Dialysis versus Hemodialysis

Of 253 patients, there were 157 (62%) patients on HD and 96 (38%) patients on PD. Patients on HD had the same mean CES-D score as patients on PD (17.2 ± 8.8 and 16.5 ± 7.9, respectively; *p*=0.55). There was no significant difference in the prevalence of depression between patients on HD and those on PD. Forty-four patients on PD had depression (45.8%), whereas 77 patients on HD had depression (49%) (*p*=0.6). Significantly more patients had poor sleep in the PD group than in the HD group (72 patients (75%) versus (vs.) 140 (89.1%), *p*=0.003), as illustrated in Table 3.

### 3.5. Quality of Life Outcomes: Comparison of Elderly versus Young Patients

Of 253 patients, there were 121 (47.8%) elderly patients (age ≥60 years) and 132 (52.2%) young patients (age <60 years).  Table 4 shows the demographic and laboratory test results between the elderly and young groups.

Sixty-five elderly patients had depression (53.7%), whereas 57 young patients had depression (43.1%) (*p*=0.09). Most of our dialysis patients had poor sleep, but this was more significant in the elderly group 109 (90%) than in the young group 103 (78%) (*p*=0.009), as illustrated in Table 4.

### 3.6. Quality of Life Outcomes: Women versus Men

Of 253 patients, there were 137 (54.1%) female patients and 116 (45.8%) male patients.  Table 5 shows the demographic and laboratory test results between the female and male groups.

The CES-D score was 17 ± 8 with a depression rate of 39%. More women had depression than men (52% vs. 25% (*p* < 0.0001; odds ratio: 3.27 (95% CI: 1.9–5.6), *p* < 0.0001)). Based on the PSQI global score, 76.5% of dialysis patients were classified as poor sleepers (women: 120 (87.6%), men: 93 (80%), *p*=0.1).

We identified three factors associated with both depression and poor sleep. First, diabetic women on dialysis had more depression and poor sleep than nondiabetic women (59% vs. 40%, *p*=0.03, and 92% vs. 79%, *p*=0.02, respectively). Second, women on HD had more depression and poor sleep than women on PD (54% vs. 46%, *p* > 0.05, and 88% vs. 65%, *p*=0.001, respectively). Third, women aged ≥60 years had more depression (59% vs. 42%, *p*=0.05) and poor sleep (92% vs. 76%, *p*=0.0008) than younger women.

There was a strong correlation between the PSQI score and CES-D score in only women (*R* = 0.67, *p* < 0.0001). There was no correlation between our studied HRQOL measures and the levels of calcium, phosphorus, PTH, Kt/V, low-density lipoprotein, or vitamin D (Table 5).

## 4. Discussion

Depression and sleep disorders represent part of the quality of life spectrum. Our study found high prevalence of depression and sleep disorder in the dialysis population in Qatar. This finding supports the hypothesis that dialysis patients have poor quality of life. Depression has been identified as the most common psychiatric illness in patients with ESRD, but its prevalence has varied widely in different studies, in different populations, and with use of different assessment tools. In our study, depressive symptoms were reported in 48% of patients, which is similar to that in other previous studies [9, 10]. This rate is substantially higher than those found in the general population, for which the rates of depression are 3–6% [11].

The comparison between patients on HD and those on PD with regard to aspects of QOL including depressive symptoms showed some degree of controversy. In 2008, differences in QOL were compared between 77 in-center HD and 58 CAPD/PD patients, and the study concluded that patients on HD experienced more compromised QOL compared to those on CAPD/PD [12]. The same results were supported later by more recent trials [13–15]. However, some other trials concluded that depression is highly more prevalent in patients on PD than in those on HD [16]. In our study, the CES-D scores were almost similar between the HD and PD groups, which significantly minimized the impact of dialysis modality on the prevalence of depression in dialysis patients.

We found that the incidence of depression is on the rise among female patients, and this has been reported elsewhere [17, 18], especially in diabetic, HD, and elderly subgroups that may be associated with a higher risk of hospitalization and poor outcomes [19]. However, no significant differences were observed in the prevalence of depression and life event variables among male and female patients in a more recent study [20]. Therefore, more studies are still needed to clarify which sex is more susceptible to depressive symptoms in the dialysis population.

Sleep disorders also are common among patients undergoing dialysis. Although variable, their prevalence has been reported to be higher than that in the general population. Sleep disorders were reported in almost 70–80% of patients on dialysis [21, 22]. In the present study, the prevalence of sleep disorders was similar to that in previous studies, but the prevalence was higher in patients on PD than in those on HD, especially in elderly and diabetic patients.

Sleep disorders have been reported to be common in diabetic patients even in those without chronic kidney diseases, and poor diabetes control is an important factor of disturbed sleep quality [23, 24]. The main risk factor for sleep disorders is depression [16, 25], which was also found to be more prevalent in diabetic patients than in other dialysis patients in the present study. Studies have revealed that there is a bidirectional relationship between sleep disorders and diabetes: diabetes may cause sleep disorders, while sleep disorders may also complicate the control of diabetes [26]; and microvascular and macrovascular complications of diabetes, such as diabetic retinopathy and cardiovascular disease, respectively, have also been linked to abnormal duration of sleep [27].

In 2011, one study compared the prevalence of different sleep disorders between patients on PD and those on HD; 227 patients with ESRD were screened using questionnaires, and the results revealed that different aspects of sleep disorders were more common in patients on PD than in those on HD [28]. More recently, Lee et al. compared the incidence of sleep disorders between 7,645 incident patients on PD and 38,225 incident patients on HD, and their results showed that patients on PD had a high risk for sleep disorders compared to those on HD [29]. The results in our study support the previous findings with regard to the higher prevalence of poor sleep in patients on PD than in those on HD; this finding may be associated with general cognitive dysfunction, as demonstrated in a recent study conducted by Zhao et al. in 2019 [30]. Therefore, patients on PD should undergo more focused, regular psychiatric evaluation that targets the identification of risk factors and therapeutic measures.

Our study revealed that older patients had more severe sleep disorders than younger patients, which is consistent with findings reported in the literature [21, 31]. These findings could be explained by concurrent geriatric comorbidities in these groups of patients. Correlations between sleep disorders and sex in patients on dialysis result in some degree of debate. Some trials concluded that sex was neither significantly correlated with the presence of sleep disorders nor with their severity [32]; however, female sex was an independent predictor of patients being poor sleepers among 164 patients on HD who had been screened by Pai et al. for sleep disorders [25]. In addition, in 227 patients on HD in Saudi Arabia, poor sleep was more significant in female patients than in male patients [33]. Our results are consistent with these previous findings, which revealed the prominence of sleep disorders in female patients on dialysis. According to the statistical analysis of our data, we identified three risk factors associated with both depression and sleep disorders in female patients on dialysis: the presence of diabetes, elderly age, and the presence of HD.

## 5. Strengths and Limitations

The strength of this study is that it is the first to assess different aspects of QOL in a considerable number of patients on dialysis in Qatar and to compare the prevalence and severity of sleep disorders and depression in patients on HD and PD. The limitations are as follows:

1. The patient population was predominantly Middle Eastern with no significant number of other ethnicities.
2. Most patients had a relatively high socioeconomic level, which limits the interpretation of results to this group of patients.
3. We did not assess the therapeutic effect of treatment even though some of the patients started medical treatment for depression and sleep disorders. Thus, we plan to follow up with these patients later in a different research study that focuses on depression and sleep disorders as the most common aspects of impaired QOL.
4. We did not cover other aspects such as chronic pain or exercise intolerance, which may have been due to insignificant prevalence of these conditions in our patients during the initial screening.

## 6. Conclusions

We performed the first study that evaluated depression and sleep disorders in patients on dialysis therapy in Qatar. Both disorders were widespread among our patients on dialysis in Qatar. Thus, it is essential for healthcare professionals to recognize different aspects of poor QOL in patients on dialysis. In addition, more studies are needed to further clarify the risk factors of depression and sleep disorders and to assess the efficacy of therapeutic measures in these patients [24, 29–33].

## Figures and Tables

**Table 1:** Demographic and clinical data of the study population.

Parameters (*N* = 253)	Means
Age	56.8 ± 14.5
Gender Male Female	116 46% 137 54%
Dialysis modality HD PD	157 62% 96 38%
Diabetic Nondiabetic	148 58.5% 105 41.5%
BMI	29.0 ± 7.1
Vit D	22.7 ± 7.9
LDL	2.6 ± 8.8
HbA1c	6.0 ± 1.9
PTH	447 ± 420
Calcium	2.24 ± 0.21
Albumin	31.3 ± 4.8
Hemoglobin	11.0 ± 1.56
Kt/V	1.75 ± 0.49
Depression score	16.9 ± 8.4
PSQI	12.1 ± 7.8

Lab results are presented as mean value ± standard deviation; BMI: body mass index; PSQI: Pittsburgh Sleep Quality Index; CES-D: Centre for Epidemiological Studies Depression (depression score); Kt/V: a number used to quantify hemodialysis and peritoneal dialysis treatment adequacy.

**Table 2:** Comparison between diabetic and nondiabetic HD patients based on different parameters.

	Diabetic *N* = 157	Nondiabetic *N* = 105	*P* value
Age	61.7 ± 11.4	49.9 ± 15.7	0.000
Gender Male Female			
BMI	31.0 ± 7.2	26.1 ± 2.2	0.001
Vit D	22.1 ± 7.6	23.5 ± 8.3	0.305
LDL	1.89 ± 0.68	3.6 ± 13.7	0.039
HbA1c	6.7 ± 1.7	4.9 ± 1.7	0.269
PTH	423.0 ± 404.2	482.6 ± 440.9	0.269
Calcium	2.25 ± 0.19	2.21 ± 0.23	0.009
Phosphorus	1.43 ± 0.43	1.55 ± 0.48	0.425
Albumin	31.1 ± 4.6	32.2 ± 4.9	0.647
Hemoglobin	10.9 ± 1.31	11.0 ± 1.8	0.029
Kt/V	1.7 ± 0.52	1.83 ± 0.44	0.597
Depression score	17.4 ± 8.7	16.3 ± 8.0	0.216
PSQI	12.8 ± 7.9	11.1 ± 7.5	0.587

**Table 3:** Comparison between patients with different dialysis modalities based on different parameters.

	HD	PD	*P* value
Age	58.4 ± 15.08	54.3 ± 13.3	0.234
BMI	29.8 ± 7.7	27.6 ± 6.0	0.007
Vit D	23.6 ± 8.0	21.3 ± 7.5	0.527
LDL	2.8 ± 11.2	2.2 ± 0.99	0.335
HbA1c	6.01 ± 2.08	6.0 ± 1.7	0.677
PTH	430.7 ± 446.5	475.0 ± 373.8	0.240
Calcium	2.17 ± 0.212	2.3 ± 0.16	0.001
Phosphorus	1.49 ± 061	1.47 ± 0.34	0.001
Albumin	31.1 ± 4.6	32.2 ± 4.9	0.032
Hemoglobin	11.09 ± 1.5	10.8 ± 1.51	0.711
Kt/V	1.55 ± 0.36	2.0 ± 0.49	0.001
Depression score	17.2 ± 8.8	16.5 ± 7.9	0.365
PSQI^∗^*∗*	10 (6–18)	9 (5–16.5)	0.02

^∗^ *∗*PSQI value is presented as median value ± interquartile range (IQR).

**Table 4:** Comparison between patients <60 and patients ≥60 years based on different parameters.

	<60 (*N* = 132)	≥60 (*N *= 121)	*P* value
BMI	27.7 ± 7.1	30.50 ± 6.9	0.978
Vit D (ng/ml)	22.41 ± 7.0	23.14 ± 8.8	0.091
LDL (mmol/l)	3.17 ± 12.2	2.02 ± 0.78	0.122
HbA1c	5.65 ± 1.8	6.53 ± 1.9	0.283
PTH (pg/ml)	498.6 ± 467.3	391.17 ± 353.7	0.004
Calcium (mmol/l)	2.24 ± 0.22	2.23 ± 0.19	0.017
Phosphorus (mmol/l)	1.60 ± 0.45	1.36 ± 0.42	00.502
Albumin (g/l)	32.0 ± 4.6	31.14 ± 4.9	0.420
Hemoglobin (g/l)	11.1 ± 1.54	10.8 ± 1.5	0.206
Kt/V	1.82 ± 0.44	1.68 ± 0.53	0.837
Depression score	15.84 ± 7.8	18.19 ± 8.98	0.071
PSQI	10.15 ± 6.8	14.27 ± 8.2	0.006

**Table 5:** Comparison between male and female patients according to different parameters.

	Male (*N* = 116)	Female (*N *= 137)	*P* value
Age	55.2 ± 14.0	58.2 ± 14.8	0.604
BMI	27.0 ± 5.5	30.7 ± 7.9	0.000
Vit D	22.3 ± 7.7	23.1 ± 8.1	0.725
HbA1c	6.05 ± 1.71	5.9 ± 2.1	0.265
LDL	3.23 ± 13.0	2.1 ± 0.86	0.089
PTH	451.5 ± 418.2	444.4 ± 422.9	0.484
Calcium	2.20 ± 0.24	2.27 ± 0.17	0.000
Phosphorus	1.53 ± 0.45	1.44 ± 0.45	0.948
Albumin	31.5 ± 4.7	31.66 ± 4.8	0.992
Hemoglobin	11.06 ± 1.54	10.95 ± 1.58	0.537
Kt/V	1.67 ± 0.44	1.82 ± 0.52	0.284
Depression score	14.7 ± 6.8	18.8 ± 9.2	0.002
PSQI	9.9 ± 6.7	14.0 ± 8.1	0.002

## Data Availability

All the data collected during the study are presented in this manuscript, and no data from the study have been or will be published separately. We attest that we will provide the data if requested according to the HMC policy.
